# Fostering of social, emotional, and cognitive skills in elementary schools: the Papilio-6to9 program

**DOI:** 10.3389/fpsyg.2025.1599946

**Published:** 2025-11-27

**Authors:** Viola Lechner, Niklas Ortelbach, Herbert Scheithauer

**Affiliations:** Department of Education and Psychology, Division Developmental Science and Applied Developmental Psychology, Freie Universität Berlin, Berlin, Germany

**Keywords:** social–emotional skills, social–emotional learning, executive functions, student-teacher relationship, emotional and behavior problems, school prevention program, developmentally appropriate prevention, classroom well-being

## Abstract

**Background:**

International guidelines emphasize the importance of holistic personal development in education, incorporating both academic and social–emotional skills. The developmentally appropriate, school-based prevention program Papilio-6to9 for students aged six to nine, implemented by trained teachers, aims at improving social, emotional, and cognitive skills and preventing behavior and emotional problems. The program differs from existing school-based prevention programs in Germany in its theoretical framework and multi-level approach targeting both students and teachers. The current study is the first to assess program effectiveness by analyzing the impact on the student-level outcomes.

**Methods:**

The pilot study employed a randomized waiting control group longitudinal design with three measurement points (pre-test, post-test, follow-up). The sample comprised 224 children (52% girls, age *M* = 7.1 years) from twelve classes. Teachers completed online questionnaires at each measurement point, assessing social–emotional problems, social skills, executive functions, and student-teacher relationship.

**Results:**

The results indicated program effects in all defined outcome areas: program participation was associated with lower problem scores, higher social skills, higher executive functions, and closer student-teacher relationship.

**Conclusion:**

The findings of the pilot evaluation indicate the effectiveness of the Papilio-6to9 program in promoting social–emotional skills and preventing emotional and behavioral problems. Future studies should aim to validate the findings with a larger sample and incorporate multiple sources of information. Limitations and recommendations for future research are discussed.

## 1 Introduction

International guidelines for school framework curriculums show the interconnectedness of academic und socialization requirements focusing on a holistic personal development to promote equitable and inclusive lifelong learning possibilities (UNESCO, International Bureau of Education (IBE), 2024; Marope, 2016). Based on the Global Sustainable Goal 4 within the Education 2030 Framework for Action, the UNESCO-IBE demands overall curriculum transformations which ensure the promotion of academic and social–emotional skills adapted to 21st-century demands (UNESCO, International Bureau of Education (IBE), 2024). While academic qualification goals are described precisely in German curriculum frameworks, educational goals often remain unclear (Siebertz-Reckzeh and Hofmann, 2017). Thus, concrete teaching recommendations and time resources to meet socialization qualification are lacking. However, due to the extensive amount of time children spend at school, elementary school environments are essential for socialization during childhood development, complementing parental influences and other childcare facilities. Research indicates that schools play a pivotal role in fostering social–emotional skills and underscores the educational environment’s efficacy in compensating skill deficiencies (Sklad et al., 2012). Thus, social–emotional skills can serve as protective factors for negative developmental trajectories (Domitrovich et al., 2017) and are the strongest long-term predictor of students’ well-being (Taylor et al., 2017).

### 1.1 Preventing emotional and behavioral problems through social–emotional learning in elementary school settings

Social–Emotional Learning (SEL) has emerged as a central component of educational programs worldwide with important positive implications for the cognitive, social, and emotional development of children and adolescents. The Collaborative for Academic, Social, and Emotional Learning (CASEL) (2024) describes intra- and interpersonal core competencies such as self-awareness, self-management, accurate interpretation of social situations, as well as communication, cooperation, and problem-solving skills as essential for a positive development. These competencies can be promoted through SEL encompassing the process of understanding and regulating emotions, fostering empathy, demonstrating prosocial behavior, establishing and maintaining positive social relationships, constructively resolving challenging situations, and making responsible decisions (Collaborative for Academic, Social, and Emotional Learning (CASEL) (2024); Weissberg et al., 2015). A growing body of evidence from meta-analyses suggests that students who participate in school-based SEL programs show a range of positive outcomes, including improved social–emotional attitudes and skills, increased prosocial behaviors, positive peer relationships, enhanced academic performance, as well as reduced emotional and behavioral problems (e.g., Cipriano et al., 2023; Corcoran et al., 2018). Although research has consistently shown that school-based SEL programs can have a positive impact on a range of outcomes, the quality of evidence varies greatly, underscoring the need for a theoretical framework and high-quality evaluation plan concerning future research (Wigelsworth et al., 2022). While numerous SEL programs are accessible, only a few programs with high-quality evaluation studies in Germany are available compared to their international counterparts. This limited availability of evidence-based SEL programs in Germany might increase the likelihood that non-evaluated interventions are also implemented in practice.

### 1.2 The role of social–emotional skills during school transitions

Normative life transitions, such as the transition from informal preschool settings to formal schooling, have the potential to influence the mental health of children and adolescents in the long term (Donaldson et al., 2023). By the vulnerable stage of school entry, children encounter new cognitive, social, and behavioral expectations (Bassok et al., 2016; Seabra-Santos et al., 2022), which require learning-related and interpersonal social skills (Purtell et al., 2020; Robson et al., 2020). Findings from literature indicate that both learning-related social skills, such as paying attention, working independently, performing well-regulated and goal-directed as well as interpersonal social skills such as problem-solving skills or being able to initiate and maintain positive peer relationships are important predictors for academic success (Robson et al., 2020; Szydlo and Farnsworth, 2023). Study results suggest that SEL interventions positively impact underlying executive functions (e.g., working memory, attentional control, impulse control, and planning), which in turn correlate with social–emotional competence and academic performance (Cortés Pascual et al., 2019; Diamond, 2013). Findings from the literature indicate a positive association between executive functions and self-regulated learning (Blair and Raver, 2015), metacognitive and motivational learning processes (Zelazo et al., 2016), as well as a positive self-image, and fewer behavioral problems (Hughes, 2011). However, children with a lack of social–emotional skills show an increased risk to experience the transition to school as destabilizing (Hart et al., 2016) which might in turn lead to negative developmental and academic outcomes (Garon-Carrier et al., 2024). Thus, fostering social, emotional, and cognitive skills in children at an early stage is crucial, as it can help to mitigate potential risk factors and promote positive developmental outcomes, including enhanced interpersonal and academic competencies (Domitrovich et al., 2017; Lechner et al., 2022).

### 1.3 The program Papilio-6to9

**Figure 1:**
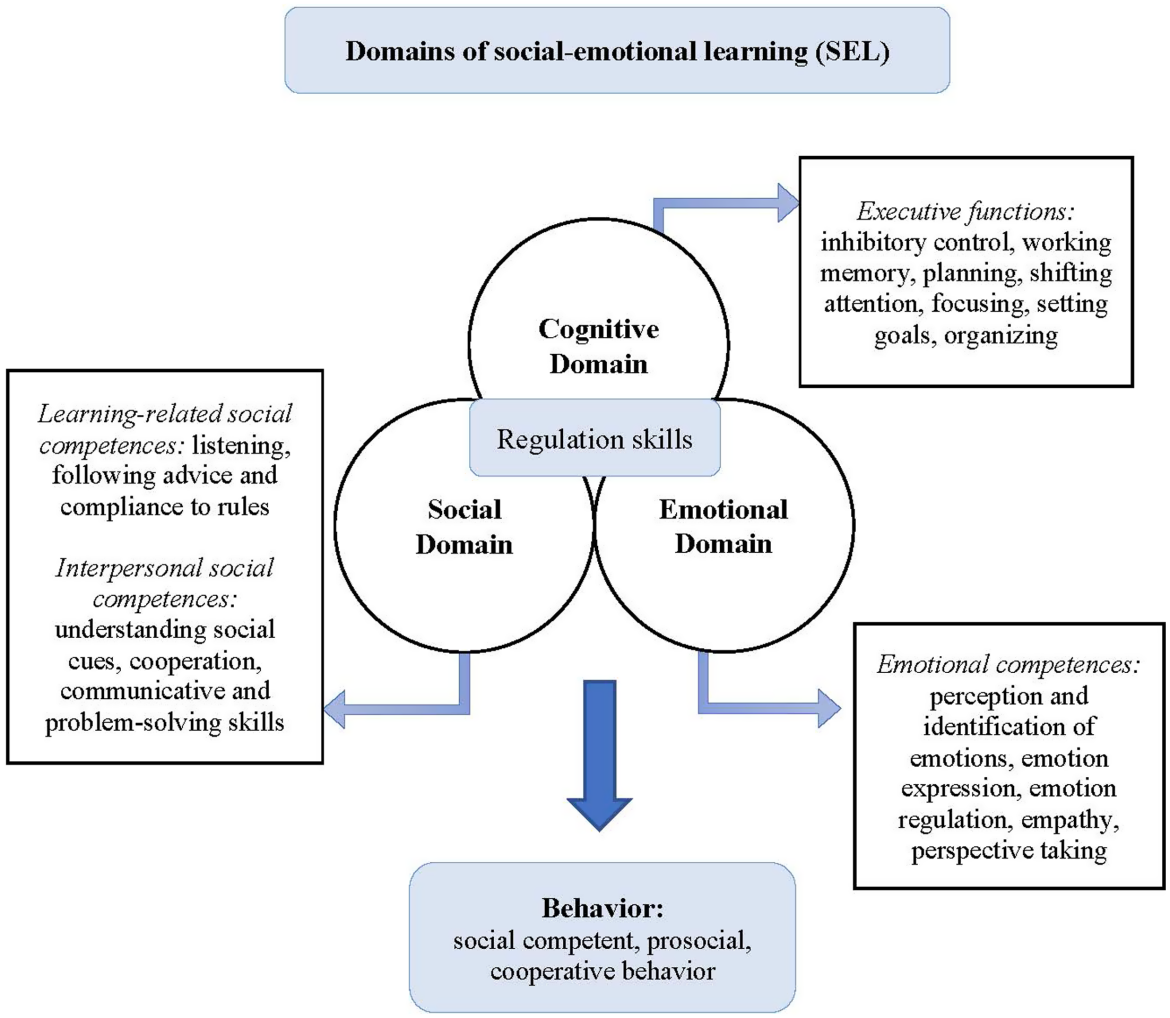
Multilevel logic model (Lechner et al., 2022, 105; cf. Campbell et al., 2016).

The program Papilio-6to9 (Lechner et al., 2020) aims to close the existing research gap by providing an effective and practice-oriented prevention strategy tailored for the German-speaking context. Papilio-6to9 (Lechner et al., 2020) is an empirically based program designed to promote social–emotional skills and prevent emotional and behavioral problems within the elementary school environment. It adheres to a manualized, structured, multi-component approach and is underpinned by a theoretical framework (Multilevel Logic Model as shown in Figure 1). The program development is based on the Intervention Mapping Approach (IMA, Bartholomew Eldredge et al., 2016) to ensure a transparent, scientific approach and a comprehensive, *a priori* need assessment (Lechner et al., 2022). The universal-selective prevention program follows principles of developmentally appropriate practice (DAP) (National Association for the Education of Young Children, 2020) which aims at reducing age-specific risk factors for emotional and behavioral problems (e.g., negative peer relationships), promoting age-specific protective factors (e.g., social–emotional and cognitive skills), and supporting children to cope with developmental tasks and normative life transitions (National Association for the Education of Young Children, 2009; Malti et al., 2009). Through play-based learning, developmentally oriented methods intentionally incorporate children’s strengths to foster self-regulation, learning motivation, and engagement (National Association for the Education of Young Children, 2020). Thus, diverse levels of demand, performance, and age-related transitions and challenges are considered. Universal-selective prevention signifies that the program targets the entire classroom population without stigmatization, irrespective of their risk status (universal), while also offering support to children at heightened risk of emotional or behavioral problems (selective). Grounded in empirical research on the social, emotional, and cognitive development of 6 to 9-year-olds, the program’s main objectives include promoting social–emotional skills, problem-solving skills, and executive functions. Furthermore, the program seeks to create a supporting learning environment by fostering positive relationships and a positive classroom climate, and by reducing emotional and behavioral problems. Additionally, the program aims at long-term improvements in academic performance by enhancing learning motivation and classroom well-being. Teachers implement the program, consisting of 27 units, each lasting 45 min, collectively with their entire class. The sequence of units is specified in the manual and does not allow for adaptation in the sequence of implementation. However, for certain units, teachers have the option to repeat and deepen the units multiple times with different exercises, depending on the age and competence level of the children. The sequence of units and topics is predetermined in the manual, as the contents of the individual units are thematically interconnected. After program implementation, supplementary guidance is provided on sustaining and integrating individual modules and exercises into regular lessons. Each unit includes an introductory and concluding ritual as well as varied methodological elements such as games, interactive stories, and individual/group exercises. In addition to the sequentially structured units, teachers have access to short exercises/games which can be integrated flexibly and independently into the ongoing school lessons. There is no specification on how often these games should be conducted. According to the Multilevel Logic Model (Figure 1), the units follow different thematic focuses:

(1) Promoting learning-related social competences (social domain): This section comprises five units which focus on fostering social competences essential for academic success. Topics covered include methods of classroom management and the introduction of procedural and behavioral class rules with the help of the Good Behavior Game (GBG, Barrish et al., 1969; Smith et al., 2021). The aim is to establish (pro)social norms, promote comprehension of rules, and regulate behavior to cultivate a positive learning environment. Recent research has underscored the efficacy of the GBG as a classroom intervention, particularly benefiting children exhibiting an increased risk of externalizing behavior problems (Leidig et al., 2022).
(2) Promoting emotional and interpersonal social competences (emotional and social domain): In order to promote the perception, identification, expression, and regulation of the basic emotions (sadness, anger, fear, and joy), the interactive story “Paula and the pixies from the box” is introduced across six units. In German mythology, a Pixie (German: Kobold) is a mostly benign, magical little creature that likes to play tricks and is known for its playful, curious nature. Within the interactive story, the children get to know four pixies, each representing one basic emotion. Subsequently, children engage with the interactive story “Paula goes to school” which deals with secondary emotions including envy, shame, guilt, and pride, as well as ambivalent and masked emotions. This thematic sequence spans a total of 16 × 45-min units. Each unit is dedicated to a comprehensive exploration of self-perception, interpersonal perception of emotions, emotion regulation, problem-solving skills, or the development of empathy- and perspective-taking skills. Collaboratively developed with the renowned German puppet theater *Augsburger Puppenkiste*, the interactive stories are complemented by a puppet show (which is available as a supplementary offering, but not a mandatory part of the program’s implementation).
(3) Promoting executive functioning (cognitive domain) and classroom well-being: Every session starts with a mindfulness exercise enhancing self-awareness and children’s attention to their emotions and body sensations in the present moment. Research on mindfulness or as Tomasulo (2020, P. 51) named it *applied consciousness* has shown many positive outcomes. For example, it is linked to improved executive functions and adaptive coping strategies (Murphy et al., 2012). Complementing the series of sequential units outlined above, supplementary games and exercises are provided for the integration into daily practice. These include activities facilitating the enhancement of executive functions, cooperative group games, and developmentally appropriate adaptions of positive psychology exercises. An example of the latter would be a positive reflection of the day as a developmentally adapted method from the Three Good Things (TGT) intervention (Seligman et al., 2005). During the exercise, the children reflect upon ‘*What went well today?*’ and think about the next day ‘*What can you do tomorrow in order to experience positive things again?*’ (Lechner et al., 2022).

#### 1.3.1 Implementation strategy

The implementation strategy follows a train-the-trainer (TTT) model that has been proven to be both effective and economical in the school setting (LaVigna et al., 2005). Trainers with a professional pedagogical background, along with prior experience in the evaluated kindergarten program Papilio-3to6 (Scheithauer and Peter, 2021), undergo a training facilitated by the program developers to familiarize themselves with the Papilio-6to9 program and the transfer of knowledge. Subsequently, teachers and pedagogical school staff (e.g., social worker) receive a comprehensive three-day course covering the manualized program implementation, supplemented with ongoing supervision during implementation. As Papilio-6to9 affects multiple levels, targeting both students and teachers, teachers receive the multi-day training that covers not only the program implementation, familiarization of materials, and fidelity of implementation, but also extends beyond these practical aspects. Specifically, the training focuses on principles of positive psychology, fostering a growth mindset and positive feedback culture to enhance learning motivation. Furthermore, the teachers reflect on their own social–emotional skills and function as role-model for social–emotional competence.

### 1.4 The present study

This study aims to conduct an initial assessment of the effectiveness of the Papilio-6to9 school-based prevention program in preventing emotional and behavioral problems while promoting social–emotional skills. Employing a waiting-control group longitudinal design with three measurement points, the study examines the impact of program participation on emotional and behavioral problems, social skills (encompassing self-control, engagement, empathy, cooperation, responsibility, and self-assertion), executive functions (encompassing working memory, inhibition, action planning, and regulation), and the quality of student-teacher relationships (levels of closeness and conflict) within intervention classes compared to those in waiting-control classes.

## 2 Methods

### 2.1 Sample

The schools were selected for inclusion in the pilot study based on the following inclusion criteria: (1) participation with at least two first-grade classes - one intervention group (IG) and one waiting control group (WCG), (2) absence of classes structured according to the so called German *JüL-Format* (cross-grade learning concept, where children attend classes in mixed-grade groups), (3) attainment of parental/guardian declaration of consent for children’s participation in the study with a minimum of 75% per class, (4) non-participation in the preceding Papilio-3to6 program. The final sample comprised twelve primary school teachers from six schools across the German federal states of Bavaria (4 schools) and North Rhine-Westphalia (2 schools). The teachers, predominantly female (92%), had an average age of 41.3 years (*SD* = 7.9) and reported an average of 12.5 years of professional experience (*SD* = 6.4). On average, teachers provided information on 19 children per class (range = 15–23). Data from 224 children (52% female, age *M* = 7.1 years) were available at the initial measurement point, with 8 children dropping out by the second measurement and an additional 7 children by the third measurement due to relocation or school transfers. Further information on the sample characteristics is provided in Table 1.

**Table 1:** Sociodemographic characteristics of the children.

Variable	IG	WCG	Total
*N*	109	115	224
Age			
*M*	7.15	7.14	7.15
*SD*	0.37	0.40	0.39
Gender, *n* (%)			
Female	58 (53%)	58 (50%)	116 (52%)
Male	51 (47%)	57 (50%)	108 (48%)
Native language, *n* (%)			
Predominantly German	56 (51%)	74 (64%)	130 (58%)
Other	53 (49%)	41 (36%)	94 (42%)
Learning disability, *n* (%)			
Yes	8 (7%)	8 (7%)	16 (7%)
No	101 (93%)	103 (93%)	204 (93%)

IG, Intervention Group; WCG, Waiting-Control Group.

### 2.2 Data collection procedure

#### 2.2.1 Recruitment strategy

A comprehensive overview of elementary schools in the German federal states of Brandenburg, North Rhine-Westphalia, and Bavaria was compiled to recruit participants for the study. These schools were contacted through an informational letter detailing the Papilio-6to9 program and were asked to express their interest or lack thereof by returning an enclosed postcard. In addition, given that the initial postal recruitment strategy yielded a low response rate, the schools were informed via school administrators, telephone canvassing, and support from school advisory centers. Schools expressing interest were subsequently provided with further information and the option to register for the study. Participating schools were incentivized with free attendance of the teacher training, ongoing supervision to ensure implementation support, free access to all program materials, and schools were invited to visit the stage play of the puppet theater Augsburger Puppenkiste that had been developed alongside the program Papilio-6to9.

#### 2.2.2 Assignment and randomization

Within each participating school, two teachers were randomly assigned to the IG or WCG, respectively. The WCG teachers were scheduled to receive the training and implement the program after the study concluded. However, due to unforeseen events such as limited availability, illnesses, and deviations from the original study design, the randomized assignments were sustained in only half of the sample (three schools). Unfortunately, the randomization protocol was not upheld at the remaining three schools, resulting in a limitation that will be addressed in the discussion section.

#### 2.2.3 Evaluation plan

The pilot effectiveness evaluation covered three measurement points: A pre-measurement (T1) was conducted before program implementation, a post-measurement (T2) was conducted immediately after program implementation (approximately 5–6 months after T1), and a follow-up measurement (T3) was conducted 3 months post-program completion. At each measurement point, the teachers provided data on the children in their class via an online questionnaire. Additionally, at T1, the IG teachers had the option to complete the questionnaire in a paper-pencil format before the training started. Constructs at the teacher level (e.g., self-efficacy beliefs, job-related stress) were assessed at T1 and T3. The present study focusses on the program effects on the child level. The study received ethical approval from the Ethics Committee of the Department of Education and Psychology at Freie Universität Berlin (reference 178/2018).

### 2.3 Instruments

All constructs investigated in this manuscript were assessed by teacher ratings, which have generally been proven to be reliable and valid (Feng et al., 2022).

#### 2.3.1 The strength and difficulties questionnaire

Teachers completed the German version of the Strength and Difficulties Questionnaire (SDQ; Klasen et al., 2003) at T1, T2, and T3. The SDQ was used to assess the children’s behavioral strengths and problems. The 25 items yield five subscales with five items per scale. These subscales are Emotional Symptoms (e.g., “many fears and easily scared”), Conduct Problems (e.g., “often fights with other children or bullies them”), Hyperactivity/Inattention (e.g., “restless, overactive, and cannot stay still for long”), Peer Relationship Problems (e.g., “rather solitary and tends to play alone”), and Prosocial Behavior (e.g., “shares readily with other children”). Teachers rated the children’s behavior on a 3-point rating scale (0 = not true, 1 = somewhat true, 2 = certainly true). A Total Difficulties Score was calculated based on the items from the first four subscales. In the sample examined, high internal consistency was observed, with Cronbach’s alpha values of 0.86 for the Total Problem Score at T1 (α_T2_ = 0.86, α_T3_ = 0.88), and 0.83 for the Prosocial Behavior subscale at T1 (α_T2_ = 0.83, α_T3_ = 0.87). Petermann et al. (2010) have confirmed the convergent and divergent validity of the SDQ in comparison with the related German questionnaire Verhaltensbeurteilungsbogen für Vorschulkinder (VBV 3–6; Döpfner et al., 1993) and reported an average reliability of Cronbach’s alpha = 0.73. Additionally, significant correlations with the Child Behavior Checklist (Achenbach, 1991) have been found (Klasen et al., 2003). The five-dimensional structure of the German-language teacher version has been validated through factor analysis (Koglin et al., 2007).

#### 2.3.2 The social skills improvement system-rating scales

The present study included the following subscales from the Social Skills Improvement System - Rating Scales (SSIS-RS; Elliott and Gresham, 2008), translated into German by the authors: Self-Control (7 items, e.g., “Stays calm when teased”), Engagement (7 items, e.g., “Participates appropriately in class”), Empathy (6 items, e.g., “Comforts others”), Cooperation (6 items, e.g., “Takes turns in conversation”), Assertion (7 items, e.g., “Says when there is a problem”), and Responsibility (6 items, e.g., “Respects the property of others”). Teachers rated the frequency of their students’ behaviors on a 3-point scale (0 = never - 2 = very often). At T1, medium to high internal consistencies (Cronbach’s alpha) ranging from 0.76 to 0.91 were obtained (α_T2_ between 0.73 and 0.91, α_T3_ between 0.74 and 0.92). A comparison between the English versions of the SSIS-RS and the Social Skills Rating System (SSRS; Gresham and Elliott, 1990) confirmed the convergent and divergent validity of the SSIS-RS. In addition, the SSIS-RS was classified as superior in terms of reliability (Gresham et al., 2011).

#### 2.3.3 Childhood executive functioning inventory

Although many neuropsychological, mostly laboratory-based, tests capture executive functions (EF), there are only a few EF rating instruments available which can be used in school settings without capturing EF though abstract and decontextualized tasks (Thorell and Catale, 2014; Zelazo et al., 2016). To consider the daily social setting, children’s EF were measured by the German translation of the Childhood Executive Functioning Inventory (CHEXI; Thorell and Nyberg, 2008). The teachers rated statements on a 5-point scale ranging from 1 = *definitely not true* to 5 = *definitely true* to record children’s difficulties in the areas of Working Memory (9 items, e.g., “Has difficulty remembering what he/she is doing, in the middle of an activity”), Planning (4 items, e.g., “Has difficulty telling a story about something that has happened so that others may easily understand“), Regulation (5 items, e.g., “Seldom seems to be able to motivate him−/herself to do something that he/she does not want to do“), and Inhibition (6 items, e.g., “Has difficulty refraining from smiling or laughing in situations where it is inappropriate”). At the first measurement point, the four scales showed high internal consistencies (Cronbach’s alpha) between 0.91 and 0.97 (α_T2_ between 0.93 and 0.97, α_T3_ between 0.92 and 0.97).

#### 2.3.4 Student-teacher relationship scale

Additionally, each teacher was asked to evaluate their individual relationship with each child using the German version of the Student-Teacher Relationship Scale (STRS) - Short Form (Pianta, 2001). The teachers rated the two subscales measuring Closeness (7 items) and Conflict (8 items) on a 5-point scale ranging from 1 = *strongly disagree* to 5 = *strongly agree*. High internal consistencies (Cronbach’s alpha) of 0.86 and 0.89 were obtained at T1 (α_T2_ = 0.88 and 0.86, α_T3_ = 0.85 and 0.89). Doumen et al. (2009) reported convergent validity comparing the assessment of closeness and conflict between teachers and students from the perspective of teachers, peers, and external observers when using the STRS.

### 2.4 Data analysis

Applying Ipsative Mean Imputation, missing values of individual items were included in the calculation of the respective scale mean values. At the scale level, there were only a small number of missing values at T1 (< 5% per scale). However, due to longitudinal dropout of children (8% from T1 to T2, 11% from T1 to T3), there were correspondingly higher dropouts of scale values at T2 and T3. Dropout analyses revealed a systematic pattern across most of the examined outcomes: At T2 and T3, younger children, as well as those with greater teacher-reported social–emotional problems (SDQ), lower social skills (SSIS-RS), lower executive functions (CHEXI), and higher scores on the conflict subscale (teacher-student relationships) at T1 were more likely to drop out. However, the dropout was not associated with the study group membership (IG or WCG) or the gender of the children. The missing values at T2 and T3 were estimated using the Expectation–Maximization (EM) algorithm implemented in SPSS. Analyses were then replicated with the imputed dataset. As these sensitivity analyses revealed no substantial differences in the results, the findings reported below are based on the analyses with listwise deletion.

Longitudinal analyses of covariance were conducted to assess the program’s effectiveness. The T2 or T3 values of the SDQ, SSIS-RS, CHEXI, and STRS were used as outcomes, controlling for the baseline value of the respective scale (T1). The group allocation (IG vs. WCG, with WCG as the reference category) was treated as a dichotomous predictor variable, whereby a significant coefficient indicated an intervention effect. Age and gender of the children were included as covariates. Standardized effect size measures and associated confidence intervals for group differences (IG vs. WCG, considering covariates) were calculated for each model (Lenth, 2022). Effect sizes below 0.05, above 0.05, and above 0.2 were classified as small, medium, and high effects, respectively, based on Kraft’s (2020) analyses of intervention effects in education.

To validate the results of the unilevel regression analyses by taking into account the nested structure of our data (children nested in school classes), we conducted multilevel analyses. Considering the small level 2 sample size, we fit the random intercept models via restricted maximum likelihood estimation with Kenward-Roger correction (McNeish and Stapleton, 2016). All analyses were conducted using SPSS version 28 and R version 4.1.2, along with R packages stats, olsrr, emmeans, lme4, and lmerTest.

## 3 Results

### 3.1 Descriptive analyses

Table 2 displays the mean values, standard deviations, and inter-correlations of the scales at T1. The age of the children showed no correlation with any scale. However, gender differences were observed with correlations in the low range: The teachers reported higher levels of social–emotional problems (SDQ Total Problem Score), as well as lower levels of Prosocial Behavior (SDQ) and social skills (SSIS-RS subscales Self-Control, Empathy, Cooperation, and Responsibility) for boys. Similarly, they reported lower levels of EF (CHEXI subscales Regulation and Inhibition) and lower levels of Closeness along with higher Conflict scores (STRS) for boys. As expected, the questionnaire scales demonstrated moderate to high correlations with each other: Scales assessing social–emotional problems, lower executive functions, or conflictual teacher-student relationships correlated positively with each other, whereas they displayed negative correlations with scales measuring social skills and teacher-student relationship closeness (Table 2).

**Table 2:** Mean values, standard deviations and correlations of the investigated scales at the first measurement point.

Variables	*N*	*M*	*SD*	1	2	3	4	5	6	7	8	9	10	11	12	13	14	15
1.	224	7.15	.40															
2.	224	1.48	0.50	.08														
3.	224	0.37	0.31	.04	.21*													
4.	218	1.54	0.49	.08	−.27*	−.62*												
5.	224	1.51	0.48	−.07	−.26*	−.71*	.64*											
6.	224	1.49	0.38	.06	−.07	−.59*	.53*	.38*										
7.	214	1.46	0.47	.07	−.24*	−.55*	.85*	.57*	.57*									
8.	224	1.49	0.45	−.05	−.22*	−.81*	.62*	.70*	.43*	.49*								
9.	224	1.29	0.36	−.01	−.04	−.33*	.33*	.19*	.50*	.39*	.26*							
10.	224	1.56	0.45	−.03	−.19*	−.80*	.68*	.75*	.55*	.58*	.87*	.38*						
11.	224	2.30	1.02	.06	.12	.69*	−.46*	−.48*	−.51*	−.45*	−.68*	−.53*	−.71*					
12.	224	2.29	1.06	.04	.13	.70*	−.51*	−.47*	−.54*	−.48*	−.67*	−.54*	−.70*	.95*				
13.	224	2.31	1.02	.05	.15*	.73*	−.52*	−.63*	−.35*	−.49*	−.81*	−.27*	−.76*	.72*	.70*			
14.	224	2.11	0.89	.01	.28*	.79*	−.67*	−.77*	−.40*	−.59*	−.83*	−.22*	−.83*	.63*	.63*	.78*		
15.	224	4.13	0.63	−.02	−.26*	−.37*	.56*	.36*	.44*	.56*	.31*	.57*	.42*	−.43*	−.47*	−.31*	−.31*	
16.	224	1.47	0.65	.07	.25*	.71*	−.66*	−.78*	−.37*	−.57*	−.68*	−.15*	−.70*	.44*	.47*	.64*	.68*	−.46*

Variables = 1. Age, 2. Gender, 3. Strengths and Difficulties Questionnaire (SDQ) Total Problem Score, 4. SDQ Prosocial Behavior Scale, 5. Social Skills Improvement System-Rating Scales (SSIS-RS) Self-Control Scale, 6. SSIS-RS Engagement Scale, 7. SSIS-RS Empathy Scale, 8. SSIS-RS Cooperation Scale, 9. SSIS-RS Assertion Scale, 10. SSIS-RS Responsibility Scale, 11. Childhood Executive Functioning Inventory (CHEXI) Working Memory Scale, 12. CHEXI Planning Scale, 13. CHEXI Regulation Scale, 14. CHEXI Inhibition Scale, 15. Student-Teacher-Relationship Scale (STRS) Closeness Scale, 16. STRS Conflict Scale. Coding Gender: 1 = Female, 2 = Male. *p < 0.05.

### 3.2 Unilevel analyses of intervention effects

#### 3.2.1 Social–emotional skills and problems

Supplementary Tables S3–S10 present the intervention effects on social–emotional problems and social skills. Controlling for the baseline values of each outcome, as well as the age and gender of the children, a significant group effect was observed for the SDQ problem score and all SSIS-RS scales at T2. Specifically, at T2, teachers in the IG reported lower problem scores (SDQ) for the children than in the WCG, *B* = −0.05, *SE* = 0.02, *p* = .041, *d* = −0.29, as well as higher scores on the SSIS-RS scales Self-Control, *B* = 0.14, *SE* = 0.04, *p* < .001, *d* = 0.54, Engagement, *B* = 0.18, *SE* = 0.04, *p* < .001, *d* = 0.69, Empathy, *B* = 0.08, *SE* = 0.04, *p* = .028, *d* = 0.32, Cooperation, *B* = 0.08, *SE* = 0.04, *p* = .029, *d* = 0.31, Assertion, *B* = 0.17, *SE* = 0.04, *p* < .001, *d* = 0.58, and Responsibility, *B* = 0.07, *SE* = 0.03, *p* = .016, *d* = 0.34. At T3, IG teachers reported higher scores for the children on the SSIS-RS scales Engagement, *B* = 0.14, *SE* = 0.04, *p* < .001, *d* = 0.54, and Assertion, *B* = 0.17, *SE* = 0.05, *p* < .001, *d* = 0.52, compared to the children of the WCG teachers. No significant group differences were found at T2 for the SDQ Prosocial Behavior scale or at T3 for both SDQ scales and the SSIS-RS scales of Self-Control, Empathy, Cooperation, and Responsibility. The effect sizes ranged from medium to high.

#### 3.2.2 Executive functions

Supplementary Tables S11–S14 illustrate the intervention effects on executive functions. Significant group effects were obtained for all CHEXI scales at T2. Controlling for the respective baseline values and the age and gender of the children, the IG teachers reported lower problem scores for Working Memory, *B* = −0.30, *SE* = 0.08, *p* < .001, *d* = −0.56, Planning, *B* = −0.35, *SE* = 0.08, *p* < .001, *d* = −0.64, Regulation, *B* = −0.18, *SE* = 0.08, *p* = .030, *d* = −0.31, and Inhibition, *B* = −0.16, *SE* = 0.06, *p* = .014, *d* = −0.35, compared to the WCG. At T3, IG teachers reported lower problem scores for Planning, *B* = −0.18, *SE* = 0.07, *p* = .011, *d* = −0.36, and Inhibition (trend), *B* = −0.11, *SE* = 0.07, *p* = .097, *d* = −0.24, relative to the WCG. No significant group effects were found for the Working Memory and Regulation scales at T3. The effect sizes were in the middle to high range.

#### 3.2.3 Student-teacher relationships

Supplementary Tables S15, S16 show the intervention effects on teacher-student relationships. A significant group effect was found for the Closeness scale at both measurement points, *B* = 0.14, *SE* = 0.05, *p* = .007, *d* = 0.38, and *B* = 0.15, *SE* = 0.06, *p* = .013, *d* = 0.36, respectively. Controlling for the baseline values, as well as the age and gender of the children, teachers in the IG reported higher Closeness scores at T2 and T3, compared to the WCG, with a high effect size. No significant group effects were obtained for the Conflict scale at either T2 or T3.

### 3.3 Multilevel analyses of intervention effects

The multilevel analyses partially replicated the unilevel regression results. With regard to the social–emotional domain, the Engagement scale of the SSIS-RS yielded a significant group effect at T2, *B* = 0.17, *SE* = 0.07, *p* = .041, and a trend for a group effect at T3, *B* = 0.13, *SE* = 0.07, *p* = .079, respectively. The Self-Control scale yielded a trend for a group effect at T2, *B* = 0.14, *SE* = 0.07, *p* = .057. Likewise, regarding the children’s EF, the Planning scale yielded a trend for a group effect at T2, *B* = −0.35, *SE* = 0.19, *p* = .100. The other significant effects delineated above were not replicated by the multilevel analyses.

## 4 Discussion

This study aimed to address the lack of research regarding multi-component, developmentally appropriate prevention programs tailored for elementary school settings in Germany. Thus, we introduced the theory-based Papilio-6to9 program and presented the initial results of the pilot study on the student level. The pilot study aimed to assess whether participation in the program leads to significant improvements in emotional and behavioral problems, learning-relevant and interpersonal social skills, executive functions, and the quality of the teacher-student relationship, thereby contributing to school-based prevention and the promotion of social–emotional skills. The study demonstrated short-term program effects on teacher assessments across all examined areas: Controlling for children’s age and gender and considering the respective baseline levels, IG teachers reported a lower total (social–emotional) problem score, higher scores on all subscales measuring social skills, lower problem scores on all subscales measuring EF, and a higher score on Closeness between students and teachers compared to the WCG at the post-test. However, no significant group differences were found on the subscales Prosocial Behavior (SDQ) and Conflict (STRS). Additionally, sustained effects were obtained at the third measurement point across all domains, specifically on the subscales Commitment and Assertion (SSIS-RS), Planning and Inhibition (CHEXI), and Closeness (STRS). Effect sizes ranged from medium to high across these measures.

The results concerning social–emotional skills and problems largely support our hypotheses. The lower problem scores obtained in children from the IG compared to the WCG, along with improvements in Self-Control, Commitment, Empathy, Cooperation, Assertion, and Responsibility suggest the effectiveness of the program in promoting social–emotional skills and preventing behavior problems. However, contrary to the expectation, no difference in prosocial behavior was found between the IG and WCG at T2. This could be due to the fact that observable prosocial behavior is a multidimensional construct reliant on various cognitive, emotional, and social skills (e.g., perspective-taking, behavioral inhibition, behavior planning, empathy, interpersonal skills), whereby its promotion requires more time, practice, and repetition than available within the given study period (Dunfield and Kuhlmeier, 2013; Eisenberg and Spinrad, 2014). At T3, enhanced values were also evident on the Engagement and Assertion subscales, which is potentially attributable to the impact of cooperative group activities aimed at promoting a positive class climate and group cohesion. The Engagement scale encompasses items such as “makes friends easily,” “interacts well with other children,” and “takes part in games or group activities,” which might serve as indicators for potential improvements in peer relationships and class climate. These aspects are designed to be integrated into everyday teaching practices also post program implementation, potentially explaining the sustained findings at T3, in contrast to other scales. The Assertion scale includes items such as “stands up for others who have been treated unfairly” or “seeks help/support from adults.” This can possibly be explained by the program effects on student-teacher relationship at T2 and T3 and by exercises on problem-solving skills.

The results concerning EF at T2 confirm the hypothesis that program participation leads to an improvement in Working Memory, Planning, Regulation, and Inhibition. At T3, while a trend towards lower problem scores on the Planning and Inhibition scales was found, the effects on Working Memory and Regulation were not sustained. Given the significant role of exercise repetition in generating neuronal changes (Zelazo et al., 2016), future program implementation and supervision should ensure the continued integration of games and exercises targeting EF into everyday teaching even after program completion.

Program participation also influenced the student-teacher relationship, evidenced by increased Closeness as hypothesized. A central component of the teacher training is the social network analysis, wherein teachers visualize the closeness and distance between children and themselves. Subsequently, the teachers reflect on how they can use their knowledge of individual children’s interests to enhance group integration and foster closer student-teacher relationships. However, no evidence of change was found in the Conflict subscale. It requires further discussion whether the translated items adequately capture the construct of “conflict,” as some items may be perceived by teachers as intrusive (e.g., “The child feels uncomfortable when I touch him/her or show physical affection,” “The child remains angry or shows resistance when I have disciplined him/her”). Overall, the majority of the results align with the hypotheses, indicating effectiveness of Papilio-6to9 across all investigated outcome areas.

At T1, no age differences were found, but small gender differences which were no longer evident at T2. In the area of social–emotional skills and problems, the total SDQ problem score was higher for boys than for girls, consistent with findings from existing literature (Koglin et al., 2007). Additionally, there is an indication that prosocial behavior tends to be more pronounced in girls, suggesting either gender-specific socialization or a methodological bias in the results. Meta-analytical evidence by Fabes and Eisenberg (2006) indicates that gender differences in prosocial behavior, favoring girls, are more common in self-report or external report assessments, whereas observational studies show no differences. Moreover, all values across recorded EF were slightly higher in girls, which also corresponds to findings from the literature. According to Zelazo et al. (2016), a few studies that have measured EF using direct assessment methods (tests) indicate that EF is slightly higher in girls (e.g., Wiebe et al., 2008). In the student-teacher relationship domain, a trend towards higher levels of perceived conflict and lower levels of closeness in boys compared to girls was found, which is also consistent with prior research (Hajovsky et al., 2017; Koepke and Harkins, 2008).

### 4.1 Limitations und future implications

#### 4.1.1 Intervention Fidelity

In this pilot study, we were not able to assess the teachers’ fidelity in terms of program implementation, although intensive efforts were made to consider the process evaluation of all measures. Specifically, this refers to the program components for which the frequency and number of repetitions was not predetermined, while the implementation of the curriculum-based units was supervised by trained Papilio-trainers and program developers. Thus, we cannot preclude that, in particular, the results of the multilevel analyses may be due to variability in program implementation between the classes. Examples include the implementation frequency of the Good Behavior Game, practices of problem-solving steps or games to foster children’s executive functions. Future studies that evaluate the program should utilize fidelity questionnaires as part of the material for every program session. On these forms, teachers would be asked to indicate the frequency of the measures they implemented. Thereby, fidelity to the program requirements can be assessed and, furthermore, dose–response-relationships uncovered.

#### 4.1.2 Sample size

While the significant results of the pilot evaluation provide initial insights into the program’s effectiveness, the findings should be interpreted with caution due to the small sample size. Therefore, a follow-up evaluation study should aim to replicate the effects with a larger sample. A larger sample would also permit the estimation of multivariate regression models that include the various outcomes that we have analyzed here in separate models. While the sample size was limited, some effects from the analysis of covariance were still apparent in the multilevel analysis (reduced to trends), suggesting that with a larger sample size, the observed effects may be more robust and potentially significant, considering the nested data structure. Given our small sample size on the classroom level, we are not able to take the third level (classrooms nested in schools) into account with the data available at this point. Future evaluations of the program with larger samples should consider a 3-level structure. This would also allow the modeling of potential influences on the school level (e.g., school climate). Furthermore, the assignment to IG and WCG should be considered at the school level instead of class level to prevent a potential exchange of information between the IG and WCG teachers. Multiple burdens on schools such as staff shortage and a lack of time complicated the acquisition of participating schools as well as the study compliance of the participating teachers. Additionally, the researchers were confronted with language barriers by communicating with parents to gather the informed consent necessary for study participation. These shortcomings should be considered in advance related to future studies.

#### 4.1.3 Dropout

The systematic dropout at T2 and T3 may affect the representativeness of the results. Especially, given the dropout of children with higher teacher-rated social–emotional problems, lower social skills, or lower executive functioning skills, it cannot be precluded that group effects might have been affected by regression to the mean effects. However, we were able to detect significant group effects on the STRS Closeness subscale which had not been affected by differential dropout. In accordance with documented feedback received from the teachers involved, the dropout seemed unrelated to group assignment (IG/WCG) and is more likely to be explained by external factors like relocation or school changes. Thus, its impact on program effectiveness is less likely.

#### 4.1.4 Randomization

Evaluating the effectiveness of school-based programs faces significant challenges, particularly when attempting to conduct randomized controlled trials (RCTs). The implementation of RCTs in school settings can be hindered by various factors, for example the need to maintain normal operating procedures (Jaycox et al., 2006). Unfortunately, due to unforeseen staff absences in the IG, the originally intended randomized assignment of teachers to the IG and WCG could not be sustained across all participating schools. This deviation from the intended randomization procedure may introduce a potential source of bias, as non-randomized assignments can lead to a selection bias and possible confounds, which may influence the outcomes. However, recent findings from a systematic review of international replication studies showed that non-randomized and quasi-experimental studies did not yield lower or biased estimates of effectiveness compared to randomized controlled trials (Waddington et al., 2023).

#### 4.1.5 Practicality

Testing the program under these real-life conditions ensures practicality and user-friendliness. Thus, process evaluation results and program material adaptions that are currently undertaken (Lechner et al., 2022) aim to reduce implementation barriers in advance to enhance feasibility.

#### 4.1.6 Source of information

To consider multiple sources of information, we initially assessed index groups (three randomly selected children per class) on their social–emotional competences at the first and third measurement occasions using the Intelligence Developmental Scales—Social Emotional Competencies (IDS-SEK; Meyer et al., 2009). However, our experience with the IDS-SEK in the pilot study revealed that the instrument has limitations and is not well-suited for capturing nuanced changes over time (Lechner et al., 2022). In future research, we plan to integrate multiple data sources, including measures that are adapted for children with limited literacy skills, which were not available in the German-speaking countries at the time of this study. Within the pilot study, the results are primarily based on teachers’ subjective views, which may introduce a bias due to the singular source of information. Additionally, teachers were informed about their study group affiliation (IG and WCG) as there was no other possibility due to the program implementation in their class. Future studies should incorporate multiple sources of information to ensure robust findings. However, teachers’ perspectives are particularly valuable for evaluating subjective changes in student behavior since a central program component is to sensitize teachers’ subjective view of the students - including improved awareness of teachers’ prejudice to reduce self-fulfilling prophecies. Furthermore, Feng et al. (2022) were able to prove that the assessment of social–emotional skills from the teacher’s perspective has the highest internal consistency and predictive power for cognitive and behavioral changes in the school setting compared to parental reports or self-reports by students.

#### 4.1.7 Conceptualization

Another challenge that all studies on social–emotional development in childhood face is the definition, conceptualization, and operationalization of the underlying constructs. Depending on the theoretical impact model, studies show major differences in the operationalization and assessment of social–emotional skills (Abrahams et al., 2019). Aligning with established models (Campbell et al., 2016), we referred to the underlying components: social competence, emotional competence, behavioral problems, self-regulation, and executive functions.

#### 4.1.8 Train-the-trainer approach

Despite criticism of the TTT approach (e.g., loss of information due to the multi-stage procedure), it can facilitate a quality-assured program rollout. Research suggests that maintaining scientific standards in teaching SEL is maintained by the TTT model (LaVigna et al., 2005). Additionally, meta-analyses have shown that teachers were able to implement SEL programs in the classroom successfully and that the involvement of extracurricular experts may not enhance effectiveness significantly (Diekstra and Gravesteijn, 2008; Durlak et al., 2022; Sklad et al., 2012).

### 4.2 Conclusion

The present pilot study suggests the overall effectiveness of Papilio-6to9 across investigated outcome areas (emotional and behavioral problems, learning-relevant and interpersonal social skills, EF, student-teacher relationship). Future research aims to validate these findings with a representative sample using a multi-level analytical approach. Additionally, current literature emphasizes the importance of a culturally and developmentally sensitive perspective of SEL and demands a critical examination of school-based SEL programs, as for example emotional expression and emotion regulation are mostly outcomes of parental education and cultural norms (Ramirez et al., 2021; Vera, 2022). Thus, evidence-based SEL programs and practices should also aim to create equitable learning environments for children and adolescents (Mahoney et al., 2021). Future adaptions of the Papilio-6to9 program are intended to consider these aspects in more detail.

## Data Availability

The datasets presented in this article are not readily available because data sharing is not applicable. Requests to access the datasets should be directed to www.developmental-science.de.

## References

[ref1] Abrahams L. Pancorbo G. Primi R. Santos D. Kyllonen P. John O. P. . (2019). Social-emotional skill assessment in children and adolescents: advances and challenges in personality, clinical, and educational contexts. Psychol. Assess. 31, 460–473. doi: 10.1037/pas0000591, PMID: 30869960

[ref2] Achenbach T. M. (1991). Manual for the child behavior checklist 4–18 and 1991 profile. Burlington: University of Vermont: Department of Psychiatry.

[ref3] Barrish H. H. Saunders M. Wolf M. M. (1969). Good behavior game: effects of individual contingencies for group consequences on disruptive behavior in a classroom. J. Appl. Behav. Anal. 2, 119–124. doi: 10.1901/jaba.1969.2-119, PMID: 16795208 PMC1311049

[ref4] Bartholomew Eldredge L. K. Markham C. M. Ruiter R. A. Fernandez M. E. Kok G. Parcel G. (2016). Planning health promotion programs: an intervention mapping approach. 4th Edn. San Francisco, CA: John Wiley & Sons.

[ref5] Bassok D. Latham S. Rorem A. (2016). Is kindergarten the new first grade? AERA Open 2:1. doi: 10.1177/2332858415616358, PMID: 26942210

[ref6] Blair C. Raver C. C. (2015). School readiness and self-regulation: a developmental psychobiological approach. Annu. Rev. Psychol. 66, 711–731. doi: 10.1146/annurev-psych-010814-015221, PMID: 25148852 PMC4682347

[ref7] Campbell S. B. Denham S. A. Howarth G. Z. Jones S. M. Whittaker J. V. Williford A. P. . (2016). Commentary on the review of measures of early childhood social and emotional development: conceptualization, critique, and recommendations. J. Appl. Dev. Psychol. 45, 19–41. doi: 10.1016/j.appdev.2016.01.008

[ref8] Cipriano C. Strambler M. J. Naples L. H. Ha C. Kirk M. Wood M. . (2023). The state of evidence for social and emotional learning: a contemporary meta-analysis of universal school-based SEL interventions. Child Dev. 94, 1181–1204. doi: 10.1111/cdev.13968, PMID: 37448158

[ref9] Collaborative for Academic, Social, and Emotional Learning (CASEL). (2024). Fundamentals of SEL. Available online at: https://casel.org/what-is-sel/ (Accessed March 13, 2024).

[ref10] Corcoran R. P. Cheung A. C. Kim E. Xie C. (2018). Effective universal school-based social and emotional learning programs for improving academic achievement: a systematic review and meta-analysis of 50 years of research. Educ. Res. Rev. 25, 56–72. doi: 10.1016/j.edurev.2017.12.001

[ref11] Cortés Pascual A. Moyano Muñoz N. Quílez Robres A. (2019). The relationship between executive functions and academic performance in primary education: review and meta-analysis. Front. Psychol. 10. doi: 10.3389/fpsyg.2019.01582, PMID: 31354585 PMC6638196

[ref12] Diamond A. (2013). Executive functions. Annu. Rev. Psychol. 64, 135–168. doi: 10.1146/annurev-psych-113011-143750 23020641 PMC4084861

[ref13] Diekstra R. Gravesteijn C.. (2008). Effectiveness of school-based social and emotional education programmes worldwide. Part one, a review of meta-analytic literature. Social and emotional education: an international analysis. Available online at: https://www.researchgate.net/profile/Rene-Diekstra-2/publication/255620397_Efectiveness_of_School-Based_Social_and_Emotional_Education_Programmes_Worldwide/links/555e0c9c08ae8c0cab2c5e7e/Efectiveness-of-School-Based-Social-and-Emotional-Education-Programmes-Worldwide.pdf (Accessed March 13, 2025).

[ref14] Domitrovich C. E. Durlak J. A. Staley K. C. Weissberg R. P. (2017). Social- emotional competence: an essential factor for promoting positive adjustment and reducing risk in school children. Child Dev. 88, 408–416. doi: 10.1111/cdev.12739, PMID: 28213889

[ref15] Donaldson C. Moore G. Hawkins J. (2023). A systematic review of school transition interventions to improve mental health and wellbeing outcomes in children and young people. School Ment. Health 15, 19–35. doi: 10.1007/s12310-022-09539-w

[ref16] Döpfner M. Berner W. Fleischmann T. Schmidt M. (1993). Verhaltensbeurteilungsbogen für Vorschulkinder (VBV 3–6) [Behavior Rating Scale for Preschool Children]. Weinheim: Beltz Test GmbH.

[ref17] Doumen S. Verschueren K. Buyse E. De Munter S. Max K. Moens L. (2009). Further examination of the convergent and discriminant validity of the student–teacher relationship scale. Infant Child Dev. 18, 502–520. doi: 10.1002/icd.635

[ref18] Dunfield K. A. Kuhlmeier V. A. (2013). Classifying prosocial behavior: helping, sharing, and comforting subtypes. Child Dev. 84, 1766–1776. doi: 10.1111/cdev.12075 23461793

[ref19] Durlak J. A. Mahoney J. L. Boyle A. E. (2022). What we know, and what we need to find out about universal, school-based social and emotional learning programs for children and adolescents: a review of meta-analyses and directions for future research. Psychol. Bull. 148, 765–782. doi: 10.1037/bul0000383

[ref20] Eisenberg N. Spinrad T. L. (2014). “Multidimensionality of prosocial behavior: rethinking the conceptualization and development of prosocial behavior” in Prosocial development: a multidimensional approach. eds. Padilla-Walker L. M. Carlo G. (New York: Oxford University Press), 17–39.

[ref21] Elliott S. N. Gresham F. M. (2008). Social skills improvement system: Rating scales manual. Bloomington, MN: Pearson Assessments.

[ref22] Fabes R. A. Eisenberg N. (2006). Meta-analyses of age and sex differences in children’s and adolescents’ prosocial behavior. In Handbook of child psychology. Social, emotional, and personality development. Eds. Eisenberg N. Damon W. Lerner R. M. (Vol. 3, 6. Aufl.). Hoboken, NJ: Wiley.

[ref23] Feng S. Han Y. Heckman J. J. Kautz T. (2022). Comparing the reliability and predictive power of child, teacher, and guardian reports of noncognitive skills. Proc. Natl. Acad. Sci. USA 119. doi: 10.1073/pnas.211399211 PMC8833216 35131849

[ref24] Garon-Carrier G. Mavungu-Blouin C. Letarte M. J. Gobeil-Bourdeau J. Fitzpatrick C. (2024). School readiness among vulnerable children: a systematic review of studies using a person-centered approach. Psicol. Reflex. Crit. 37:16. doi: 10.1186/s41155-024-00298-y, PMID: 38630214 PMC11024069

[ref25] Gresham F. M. Elliott S. N. (1990). Social skills rating system: manual. Minneapolis, MN: American guidance service.

[ref26] Gresham F. M. Elliott S. N. Vance M. J. Cook C. R. (2011). Comparability of the social skills rating system to the social skills improvement system: content and psychometric comparisons across elementary and secondary age levels. Sch. Psychol. Q. 26, 27–44. doi: 10.1037/a0022662

[ref27] Hajovsky D. B. Mason B. A. McCune L. A. (2017). Teacher-student relationship quality and academic achievement in elementary school: a longitudinal examination of gender differences. J. Sch. Psychol. 63, 119–133. doi: 10.1016/j.jsp.2017.04.001, PMID: 28633935

[ref28] Hart K. C. Graziano P. A. Kent K. M. Kuriyan A. Garcia A. Rodriguez M. . (2016). Early intervention for children with behavior problems in summer settings: results from a pilot evaluation in head start preschools. J. Early Interv. 38, 92–117. doi: 10.1177/1053815116645923

[ref29] Hughes C. (2011). Changes and challenges in 20 years of research into the development of executive functions. Infant Child Dev. 20, 251–271. doi: 10.1002/icd.736

[ref30] Jaycox L. H. McCaffrey D. F. Ocampo B. W. Shelley G. A. Blake S. M. Peterson D. J. . (2006). Challenges in the evaluation and implementation of school-based prevention and intervention programs on sensitive topics. Am. J. Eval. 27, 320–336. doi: 10.1177/1098214006291010

[ref31] Klasen H. Woerner W. Rothenberger A. Goodman R. (2003). Die deutsche Fassung des strengths and difficulties questionnaire (SDQ-Deu): Übersicht und Bewertung erster Validierungs- und Normierungsbefunde [German version of the strengths and difficulties questionnaire (SDQ-Deu): overview over first validation and normative studies]. Prax. Kinderpsychol. Kinderpsychiatr. 52, 491–502. 14526759

[ref32] Koepke M. F. Harkins D. A. (2008). Conflict in the classroom: gender differences in the teacher–child relationship. Early Educ. Dev. 19, 843–864. doi: 10.23668/psycharchives.11726

[ref33] Koglin U. Barquero B. Mayer H. Scheithauer H. Petermann F. (2007). Deutsche version des strengths and difficulties questionnaire (SDQ-Deu) [German version of the strengths and difficulties questionnaire (SDQ-Deu)]. Diagnostica 53, 175–183. doi: 10.1026/0012-1924.53.4.175

[ref34] Kraft M. A. (2020). Interpreting effect sizes of education interventions. Educ. Res. 49, 241–253. doi: 10.3102/0013189X20912798

[ref35] LaVigna G. W. Christian L. Willis T. J. (2005). Developing behavioural services to meet defined standards within a national system of specialist education services. Pediatr. Rehabil. 8, 144–155. doi: 10.1080/13638490400024036, PMID: 16089255

[ref36] Lechner V. Ortelbach N. Peter C. Scheithauer H. (2022). Developmentally appropriate prevention of behavior and emotional problems and fostering social and emotional skills in elementary school – overview of program theory and measures of the preventive intervention program Papilio-6to9. Int. J. Dev. Sci. 16, 99–118. doi: 10.3233/DEV-220335

[ref37] Lechner V. Peter C. Ortelbach N. Adam-Gutsch D. Scheithauer H. (2020). Papilio-6bis9 - Ein Präventionsprogramm zur Förderung sozial-emotionaler Kompetenzen in der Grundschule. Praxisordner für LehrerInnen und pädagogische Fachkräfte [Papilio-6to9 – A prevention program to promote social-emotional competencies in elementary school. Practical guide for teachers and educational staff]. Augsburg, Germany: Papilio gGmbH.

[ref38] Leidig T. Casale G. Wilbert J. Hennemann T. Volpe R. J. Briesch A. M. . (2022). Individual, generalized, and moderated effects of the good behavior game on t-risk primary school students: a multilevel multiple baseline study using behavioral progress monitoring. Front. Educ. 7:917138. doi: 10.3389/feduc.2022.917138

[ref39] Lenth R. V. (2022). Emmeans: estimated marginal means, aka least-squares means (R package version 1.7.2) [computer software]. The Comprehensive R Archive Network. Available online at: https://CRAN.R-project.org/package=emmeans.

[ref40] Mahoney J. L. Weissberg R. P. Greenberg M. T. Dusenbury L. Jagers R. J. Niemi K. . (2021). Systemic social and emotional learning: promoting educational success for all preschool to high school students. Am. Psychol. 76, 1128–1142. doi: 10.1037/amp0000701, PMID: 33030926

[ref41] Malti T. Noam G. G. Scheithauer H. (2009). Developmentally appropriate prevention of aggression—developmental science as an integrative framework. Eur. J. Dev. Sci. 3, 215–217. doi: 10.3233/DEV-2009-3301

[ref42] Marope P. T. M. (2016). Quality and development-relevant education and learning: setting the stage for the education 2030 agenda. Prospects 46, 1–33. doi: 10.1007/s11125-016-9387-0

[ref43] McNeish D. Stapleton L. M. (2016). Modeling clustered data with very few clusters. Multivar. Behav. Res. 51, 495–518. doi: 10.1080/00273171.2016.1167008, PMID: 27269278

[ref44] Meyer C. S. Hagmann-von Arx P. Grob A. (2009). Die intelligence and development scale - Sozial-Emotionale Kompetenz (IDS-SEK) - Psychometrische Eigenschaften eines tests zur Erfassung sozial-emotionaler Fähigkeiten [the intelligence and development scale of social-emotional competence (IDS-SEK) - psychometric properties of a test for assessing social-emotional abilities]. Diagnostica 55, 234–244. doi: 10.1026/0012-1924.55.4.234

[ref45] Murphy M. J. Mermelstein L. C. Edwards K. M. Gidycz C. A. (2012). The benefits of dispositional mindfulness in physical health: a longitudinal study of female college students. J. Am. Coll. Heal. 60, 341–348. doi: 10.1080/07448481.2011.629260, PMID: 22686356

[ref46] National Association for the Education of Young Children. (2020). Principles of child development and learning and implications that inform practice. Developmentally appropriate practice (DAP) position statement. Available online at: https://www.naeyc.org/resources/position-statements/dap/contents (Accessed July 15, 2024).

[ref47] National Association for the Education of Young Children. (2009). Developmentally appropriate practice in early childhood programs serving children from birth through age 8. A position statement of the National Association for the education of young children. Available online at: http://www.naeyc.org/files/naeyc/file/positions/PSDAP.pdf (Accessed July 15, 2024).

[ref48] Petermann U. Petermann F. Schreyer I. (2010). The German strengths and difficulties questionnaire (SDQ). Eur. J. Psychol. Assess. 26, 256–262. doi: 10.1027/1015-5759/a000034

[ref49] Pianta R. C. (2001). Student–teacher relationship scale–short form. Lutz, FL, USA: Psychological Assessment Resources.

[ref50] Purtell K. M. Valauri A. Rhoad-Drogalis A. Jiang H. Justice L. M. Lin T. J. . (2020). Understanding policies and practices that support successful transitions to kindergarten. Early Child Res. Q. 52, 5–14. doi: 10.1016/j.ecresq.2019.09.003

[ref51] Ramirez T. Brush K. Raisch N. Bailey R. Jones S. M. (2021). Equity in social emotional learning programs: a content analysis of equitable practices in PreK-5 SEL programs. Front. Educ. 6:679467. doi: 10.3389/feduc.2021.679467

[ref52] Robson D. A. Allen M. S. Howard S. J. (2020). Self-regulation in childhood as a predictor of future outcomes: a meta-analytic review. Psychol. Bull. 146, 324–354. doi: 10.1037/bul0000227, PMID: 31904248

[ref53] Scheithauer H. Peter C. (2021). Papilio-3bis6. Förderungsozial-emotionaler Kompetenz - Prävention von Verhaltens- und emotionalenProblemen. Ein Programm für Kindertagesstätten. Theorie und Grundlagen. 1.Neuauflage [Papilio-3to6. Fostering of social-emotional competencies and universal prevention of behavior and emotional problems in early childhood education and care settings- theory and basics]. Augsburg, Germany: Papilio Verlag.

[ref54] Seabra-Santos M. Major S. Patras J. Pereira M. Pimentel M. Baptista E. . (2022). Transition to primary school of children in economic disadvantage: does a preschool teacher training program make a difference? Early Child. Educ. J. 50, 1071–1081. doi: 10.1007/s10643-021-01240-y

[ref55] Seligman M. E. P. Steen T. A. Park N. Peterson C. (2005). Positive psychology Progress: empirical validation of interventions. Am. Psychol. 60, 410–421. doi: 10.1037/0003-066X.60.5.410, PMID: 16045394

[ref56] Siebertz-Reckzeh K. Hofmann H. (2017). “Sozialisationsinstanz Schule [school as a socialization institution]” in Lehrer-Schüler-Interaktion. Inhaltsfelder, Forschungsperspektiven und methodische Zugänge [teacher-student-interaction. Topics, research perspectives, and methodological approaches]. ed. Schweer M. K. W. (Wiesbaden: Springer VS), 3–26.

[ref57] Sklad M. Diekstra R. Ritter M. D. Ben J. Gravesteijn C. (2012). Effectiveness of school-based universal social, emotional, and behavioral programs: do they enhance students’ development in the area of skill, behavior, and adjustment? Psychol. Sch. 49, 892–909. doi: 10.1002/pits.21641

[ref58] Smith S. Barajas K. Ellis B. Moore C. McCauley S. Reichow B. (2021). A meta-analytic review of randomized controlled trials of the good behavior game. Behav. Modif. 45, 641–666. doi: 10.1177/0145445519878670, PMID: 31578077

[ref59] Szydlo T. M. Farnsworth E. M. (2023). Impact of kindergarten transition practices in promoting positive behavioral school readiness skills. Perspect. Early Child. Psychol. Educ. 7:10. doi: 10.58948/2834-8257.1039

[ref60] Taylor R. D. Oberle E. Durlak J. A. Weissberg R. P. (2017). Promoting positive youth development through school-based social and emotional learning interventions: a meta-analysis of follow-up effects. Child Dev. 88, 1156–1171. doi: 10.1111/cdev.12864, PMID: 28685826

[ref61] Thorell L. B. Catale C. (2014). “The assessment of executive functioning using the childhood executive functioning inventory (CHEXI)” in Handbook of executive functioning. eds. Goldstein S. Naglieri J. (New York: Springer), 359–366.

[ref62] Thorell L. B. Nyberg L. (2008). The childhood executive functioning inventory (CHEXI): a new rating instrument for parents and teachers. Dev. Neuropsychol. 33, 536–552. doi: 10.1080/87565640802101516, PMID: 18568903

[ref63] Tomasulo D. (2020). Learned hopefulness: The power of positivity to overcome depression. Oakland, CA, USA: New Harbinger Publications.

[ref64] UNESCO, International Bureau of Education (IBE). (2024). Vision and Mission. Available online at: https://www.ibe.unesco.org/en/about-us/vision-and-mission (Accessed November 4, 2024).

[ref65] Vera E. M. (2022). Social emotional learning and cultural relevancy: real world challenges. Prev. Sch. Fail. 67, 233–245. doi: 10.1080/1045988X.2022.2109565

[ref66] Waddington H. S. Villar P. F. Valentine J. C. (2023). Can non-randomised studies of interventions provide unbiased effect estimates? A systematic review of international replication studies. Eval. Rev. 47, 563–593. doi: 10.1177/0193841X221116721 36047928 PMC10186563

[ref67] Weissberg R. P. Durlak J. A. Domitrovich C. E. Gullotta T. P. (2015). “Social and emotional learning: past, present, and future” in Handbook of social and emotional learning: Research and practice. eds. Durlak J. A. Domitrovich C. E. Weissberg R. P. Gullotta T. P. (New York: The Guilford Press), 3–19.

[ref68] Wiebe S. A. Epsy K. A. Charak D. (2008). Using confirmatory factor analysis to understand executive control in preschool children: I. Latent structure. Dev. Psychol. 44, 575–587. doi: 10.1037/0012-1649.44.2.575 18331145

[ref69] Wigelsworth M. Verity L. Mason C. Qualter P. Humphrey N. (2022). Social and emotional learning in primary schools: a review of the current state of evidence. Br. J. Educ. Psychol. 92, 898–924. doi: 10.1111/bjep.12480, PMID: 34921555 PMC9540263

[ref70] Zelazo P. D. Blair C. B. Willoughby M. T. (2016). Executive function: implications for education. NCER 2017–2000. National Center for Education Research. Available online at: https://ies.ed.gov/sites/default/files/ncer/document/2024/11/20172000.pdf (Accessed March 3, 2024).

