# Supplementary material for: Fostering of social, emotional, and cognitive skills in elementary schools: the Papilio-6to9 program
Source: Front Psychol. 2025 Nov 27;16:1599946. doi: 10.3389/fpsyg.2025.1599946 (PMC12695554; doi:10.3389/fpsyg.2025.1599946)
Supplement: Supplementary file 1 [file Table_1.DOCX]

| **Table 3**  *Strengths and Difficulties Questionnaire* (*SDQ) Total Problem Score, Results of the Longitudinal Analyses of Covariance at the Second and Third Measurement Point* | | | | | | |
| --- | --- | --- | --- | --- | --- | --- |
|  | **T2** | | | **T3** | | |
| Variables | *B (SE)* | 95% CI | *p* | *B (SE)* | 95% CI | *p* |
| (Constant) | 0.01 (0.20) | -0.39 – 0.41 | 0.978 | 0.09 (0.23) | -0.36 – 0.53 | 0.699 |
| Baseline | 0.90 (0.04) | 0.82 – 0.97 | **<0.001** | 0.85 (0.04) | 0.76 – 0.93 | **<0.001** |
| Study Group | -0.05 (0.02) | -0.09 – -0.00 | **0.041** | -0.02 (0.02) | -0.06 – 0.03 | 0.518 |
| Gender | -0.03 (0.02) | -0.08 – 0.01 | 0.151 | -0.03 (0.03) | -0.08 – 0.02 | 0.200 |
| Age | 0.01 (0.03) | -0.05 – 0.07 | 0.745 | -0.00 (0.03) | -0.07 – 0.06 | 0.920 |
| *N* | 206 | | | 200 | | |
| *R*^2^ / *R*^2^ adjusted | 0.741 / 0.736 | | | 0.668 / 0.661 | | |
| *d* [95% CI] | - 0.287 [-0.564 – -0.010] | | | - 0.092 [-0.372 – 0.188] | | |
| *Note.* CI = Confidence Interval, Coding Study Group: 1 = Intervention, 0 = Waiting Control Group (Reference), Coding Gender: 1 = Female, 2 = Male. | | | | | | |

| **Table 4**  *Strengths and Difficulties Questionnaire* (*SDQ) Subscale Prosocial Behavior, Results of the Longitudinal Analyses of Covariance at the Second and Third Measurement Point* | | | | | | |
| --- | --- | --- | --- | --- | --- | --- |
|  | **T2** | | | **T3** | | |
| *Variables* | *B (SE)* | 95% CI | *p* | *B (SE)* | 95% CI | *p* |
| (Constant) | 0.08 (0.35) | -0.61 – 0.77 | 0.812 | 0.59 (0.39) | -0.17 – 1.36 | 0.128 |
| Baseline | 0.68 (0.04) | 0.59 – 0.76 | **<0.001** | 0.70 (0.05) | 0.61 – 0.79 | **<0.001** |
| Study Group | 0.04 (0.04) | -0.04 – 0.12 | 0.295 | 0.02 (0.04) | -0.07 – 0.10 | 0.664 |
| Gender | 0.01 (0.04) | -0.07 – 0.09 | 0.824 | -0.01 (0.04) | -0.09 – 0.08 | 0.885 |
| Age | 0.06 (0.05) | -0.03 – 0.16 | 0.193 | -0.00 (0.05) | -0.11 – 0.10 | 0.932 |
| *N* | 201 | | | 195 | | |
| *R*^2^ / *R*^2^ adjusted | 0.605 / 0.597 | | | 0.574 / 0.565 | | |
| *d* [95% CI] | 0.151 [-0.132 – 0.433] | | | 0.063 [-0.224 – 0.351] | | |
| *Note.* CI = Confidence Interval, Coding Study Group: 1 = Intervention, 0 = Waiting Control Group (Reference), Coding Gender: 1 = Female, 2 = Male. | | | | | | |

| **Table 5**  *Social Skills Improvement System-Rating Scales (SSIS-RS)* *Subscale Self-Control, Results of the Longitudinal Analyses of Covariance at the Second and Third Measurement Time Points* | | | | | | |
| --- | --- | --- | --- | --- | --- | --- |
|  | **T2** | | | **T3** | | |
| Variables | *B (SE)* | 95% CI | *p* | *B (SE)* | 95% CI | *p* |
| (Constant) | 0.55 (0.35) | -0.13 – 1.24 | 0.114 | 0.67 (0.39) | -0.11 – 1.44 | 0.090 |
| Baseline | 0.63 (0.04) | 0.55 – 0.71 | **<0.001** | 0.69 (0.05) | 0.60 – 0.78 | **<0.001** |
| Study Group | 0.14 (0.04) | 0.07 – 0.21 | **<0.001** | 0.01 (0.04) | -0.07 – 0.10 | 0.721 |
| Gender | -0.04 (0.04) | -0.12 – 0.03 | 0.248 | -0.07 (0.04) | -0.15 – 0.02 | 0.117 |
| Age | 0.02 (0.05) | -0.07 – 0.11 | 0.694 | 0.00 (0.05) | -0.10 – 0.11 | 0.988 |
| *N* | 205 | | | 200 | | |
| *R*^2^ / *R*^2^ adjusted | 0.598 / 0.590 | | | 0.567 / 0.559 | | |
| *d* [95% CI] | 0.542 [0.261 – 0.823] | | | 0.051 [-0.229 – 0.330] | | |
| *Note.* CI = Confidence Interval, Coding Study Group: 1 = Intervention, 0 = Waiting Control Group (Reference), Coding Gender: 1 = Female, 2 = Male. | | | | | | |

| **Table 6**  *Social Skills Improvement System-Rating Scales (SSIS-RS)* *Subscale Engagement, Results of the Longitudinal Analyses of Covariance at the Second and Third Measurement Point* | | | | | | |
| --- | --- | --- | --- | --- | --- | --- |
|  | **T2** | | | **T3** | | |
| Variables | *B (SE)* | 95% CI | *p* | *B (SE)* | 95% CI | *p* |
| (Constant) | 0.85 (0.34) | 0.18 – 1.51 | **0.013** | 0.60 (0.34) | -0.06 – 1.26 | 0.075 |
| Baseline | 0.64 (0.05) | 0.54 – 0.73 | **<0.001** | 0.65 (0.05) | 0.56 – 0.75 | **<0.001** |
| Study Group | 0.18 (0.04) | 0.10 – 0.25 | **<0.001** | 0.14 (0.04) | 0.07 – 0.21 | **<0.001** |
| Gender | -0.01 (0.04) | -0.08 – 0.07 | 0.873 | -0.00 (0.04) | -0.07 – 0.07 | 0.989 |
| Age | -0.04 (0.05) | -0.13 – 0.05 | 0.381 | -0.01 (0.05) | -0.10 – 0.08 | 0.813 |
| *N* | 205 | | | 200 | | |
| *R*^2^ / *R*^2^ adjusted | 0.523 / 0.513 | | | 0.522 / 0.512 | | |
| *d* [95% CI] | 0.685 [0.399 – 0.971] | | | 0.542 [0.256 – 0.829] | | |
| *Note.* CI = Confidence Interval, Coding Study Group: 1 = Intervention, 0 = Waiting Control Group (Reference), Coding Gender: 1 = Female, 2 = Male. | | | | | | |

| **Table 7**  *Social Skills Improvement System-Rating Scales (SSIS-RS)* *Subscale Empathy, Results of the Longitudinal Analyses of Covariance at the Second and Third Measurement Point* | | | | | | |
| --- | --- | --- | --- | --- | --- | --- |
|  | **T2** | | | **T3** | | |
| Variables | *B (SE)* | 95% CI | *p* | *B (SE)* | 95% CI | *p* |
| (Constant) | 0.26 (0.35) | -0.43 – 0.96 | 0.453 | 0.92 (0.44) | 0.05 – 1.80 | **0.039** |
| Baseline | 0.65 (0.04) | 0.57 – 0.74 | **<0.001** | 0.60 (0.05) | 0.49 – 0.70 | **<0.001** |
| Study Group | 0.08 (0.04) | 0.01 – 0.16 | **0.028** | 0.01 (0.05) | -0.09 – 0.10 | 0.895 |
| Gender | -0.10 (0.04) | -0.18 – -0.03 | **0.009** | -0.14 (0.05) | -0.24 – -0.04 | **0.007** |
| Age | 0.06 (0.05) | -0.04 – 0.16 | 0.210 | -0.00 (0.06) | -0.13 – 0.12 | 0.937 |
| *N* | 196 | | | 191 | | |
| *R*^2^ / *R*^2^ adjusted | 0.618 / 0.610 | | | 0.464 / 0.452 | | |
| *d* [95% CI] | 0.317 [0.032 – 0.601] | | | 0.019 [-0.267 – 0.306] | | |
| *Note.* CI = Confidence Interval, Coding Study Group: 1 = Intervention, 0 = Waiting Control Group (Reference), Coding Gender: 1 = Female, 2 = Male. | | | | | | |

| **Table 8**  *Social Skills Improvement System-Rating Scales (SSIS-RS)* *Subscale Cooperation, Results of the Longitudinal Analyses of Covariance at the Second and Third Measurement Point* | | | | | | |
| --- | --- | --- | --- | --- | --- | --- |
|  | **T2** | | | **T3** | | |
| Variables | *B (SE)* | 95% CI | *p* | *B (SE)* | 95% CI | *p* |
| (Konstante) | 0.22 (0.36) | -0.49 – 0.92 | 0.542 | 0.16 (0.36) | -0.55 – 0.87 | 0.650 |
| Baseline | 0.86 (0.04) | 0.77 – 0.94 | **<0.001** | 0.89 (0.05) | 0.80 – 0.98 | **<0.001** |
| Study Group | 0.08 (0.04) | 0.01 – 0.15 | **0.029** | 0.05 (0.04) | -0.02 – 0.13 | 0.153 |
| Gender | -0.03 (0.04) | -0.11 – 0.04 | 0.421 | -0.03 (0.04) | -0.11 – 0.05 | 0.438 |
| Age | 0.00 (0.05) | -0.09 – 0.10 | 0.948 | 0.01 (0.05) | -0.09 – 0.10 | 0.894 |
| *N* | 205 | | | 200 | | |
| *R*^2^ / *R*^2^ adjusted | 0.679 / 0.672 | | | 0.680 / 0.674 | | |
| *d* [95% CI] | 0.308 [0.030 – 0.586] | | | 0.204 [-0.077 – 0.485] | | |
| *Note.* CI = Confidence Interval, Coding Study Group: 1 = Intervention, 0 = Waiting Control Group (Reference), Coding Gender: 1 = Female, 2 = Male. | | | | | | |

| **Table 9**  *Social Skills Improvement System-Rating Scales (SSIS-RS)* *Subscale Assertion, Results of the Longitudinal Analyses of Covariance at the Second and Third Measurement Point* | | | | | | |
| --- | --- | --- | --- | --- | --- | --- |
|  | **T2** | | | **T3** | | |
| Variables | *B (SE)* | 95% CI | *p* | *B (SE)* | 95% CI | *p* |
| (Constant) | -0.24 (0.39) | -1.01 – 0.53 | 0.538 | -0.19 (0.43) | -1.03 – 0.66 | 0.665 |
| Baseline | 0.80 (0.06) | 0.68 – 0.91 | **<0.001** | 0.77 (0.06) | 0.64 – 0.89 | **<0.001** |
| Study Group | 0.17 (0.04) | 0.09 – 0.25 | **<0.001** | 0.17 (0.05) | 0.08 – 0.26 | **<0.001** |
| Gender | -0.03 (0.04) | -0.11 – 0.05 | 0.493 | -0.06 (0.05) | -0.15 – 0.03 | 0.201 |
| Age | 0.07 (0.05) | -0.03 – 0.18 | 0.177 | 0.08 (0.06) | -0.04 – 0.19 | 0.182 |
| *N* | 205 | | | 200 | | |
| *R*^2^ / *R*^2^ adjusted | 0.500 / 0.490 | | | 0.440 / 0.428 | | |
| *d* [95% CI] | 0.582 [0.296 – 0.868] | | | 0.524 [0.236 – 0.813] | | |
| *Note.* CI = Confidence Interval, Coding Study Group: 1 = Intervention, 0 = Waiting Control Group (Reference), Coding Gender: 1 = Female, 2 = Male. | | | | | | |

| **Table 10**  *Social Skills Improvement System-Rating Scales (SSIS-RS)* *Subscale Responsibility, Results of the Longitudinal Analyses of Covariance at the Second and Third Measurement Point* | | | | | | |
| --- | --- | --- | --- | --- | --- | --- |
|  | **T2** | | | **T3** | | |
| Variables | *B (SE)* | 95% CI | *p* | *B (SE)* | 95% CI | *p* |
| (Constant) | 0.63 (0.28) | 0.07 – 1.18 | **0.026** | 0.39 (0.31) | -0.23 – 1.00 | 0.222 |
| Baseline | 0.79 (0.03) | 0.72 – 0.85 | **<0.001** | 0.79 (0.04) | 0.71 – 0.86 | **<0.001** |
| Study Group | 0.07 (0.03) | 0.01 – 0.13 | **0.016** | 0.01 (0.03) | -0.05 – 0.08 | 0.660 |
| Gender | -0.08 (0.03) | -0.14 – -0.02 | **0.010** | -0.06 (0.03) | -0.13 – 0.01 | 0.076 |
| Age | -0.02 (0.04) | -0.10 – 0.05 | 0.521 | 0.01 (0.04) | -0.07 – 0.10 | 0.778 |
| *N* | 205 | | | 200 | | |
| *R*^2^ / *R*^2^ adjusted | 0.754 / 0.749 | | | 0.694 / 0.688 | | |
| *d* [95% CI] | 0.341 [0.062 – 0.619] | | | 0.063 [-0.218 – 0.343] | | |
| *Note.* CI = Confidence Interval, Coding Study Group: 1 = Intervention, 0 = Waiting Control Group (Reference), Coding Gender: 1 = Female, 2 = Male. | | | | | | |

| **Table 11**  *Childhood Executive Functioning Inventory (CHEXI)* *Subscale Working Memory, Results of the Longitudinal Analyses of Covariance at the Second and Third Measurement Point* | | | | | | |
| --- | --- | --- | --- | --- | --- | --- |
|  | **T2** | | | **T3** | | |
| Variables | *B (SE)* | 95% CI | *p* | *B (SE)* | 95% CI | *p* |
| (Constant) | -0.48 (0.70) | -1.85 – 0.90 | 0.496 | 0.19 (0.67) | -1.14 – 1.51 | 0.781 |
| Baseline | 0.92 (0.04) | 0.85 – 1.00 | **<0.001** | 0.87 (0.04) | 0.79 – 0.94 | **<0.001** |
| Study Group | -0.30 (0.08) | -0.45 – -0.15 | **<0.001** | -0.11 (0.07) | -0.25 – 0.04 | 0.143 |
| Gender | -0.14 (0.08) | -0.29 – 0.01 | 0.070 | -0.12 (0.07) | -0.26 – 0.03 | 0.118 |
| Age | 0.14 (0.10) | -0.06 – 0.33 | 0.164 | 0.03 (0.09) | -0.16 – 0.21 | 0.790 |
| *N* | 205 | | | 200 | | |
| *R*^2^ / *R*^2^ adjusted | 0.745 / 0.740 | | | 0.728 / 0.722 | | |
| *d* [95% CI] | -0.563 [-0.845 – -0.281] | | | -0.209 [-0.490 – 0.072] | | |
| *Note.* CI = Confidence Interval, Coding Study Group: 1 = Intervention, 0 = Waiting Control Group (Reference), Coding Gender: 1 = Female, 2 = Male. | | | | | | |

| **Table 12**  *Childhood Executive Functioning Inventory (CHEXI)* *Subscale Planning, Results of the Longitudinal Analyses of Covariance at the Second and Third Measurement Point* | | | | | | |
| --- | --- | --- | --- | --- | --- | --- |
|  | **T2** | | | **T3** | | |
| Variables | *B (SE)* | 95% CI | *p* | *B (SE)* | 95% CI | *p* |
| (Constant) | -1.15 (0.70) | -2.54 – 0.24 | 0.104 | -0.18 (0.66) | -1.48 – 1.11 | 0.781 |
| Baseline | 0.89 (0.04) | 0.82 – 0.97 | **<0.001** | 0.87 (0.04) | 0.80 – 0.94 | **<0.001** |
| Study Group | -0.35 (0.08) | -0.50 – -0.20 | **<0.001** | -0.18 (0.07) | -0.32 – -0.04 | **0.011** |
| Gender | -0.09 (0.08) | -0.24 – 0.06 | 0.239 | -0.10 (0.07) | -0.25 – 0.04 | 0.153 |
| Age | 0.23 (0.10) | 0.03 – 0.43 | **0.021** | 0.07 (0.09) | -0.11 – 0.26 | 0.434 |
| *N* | 205 | | | 200 | | |
| *R*^2^ / *R*^2^ adjusted | 0.744 / 0.739 | | | 0.749 / 0.744 | | |
| *d* [95% CI] | -0.642 [-0.925 – -0.359] | | | -0.363 [-0.645 – -0.082] | | |

*Note.* CI = Confidence Interval, Coding Study Group: 1 = Intervention, 0 = Waiting Control Group (Reference), Coding Gender: 1 = Female, 2 = Male.

| **Table 13**  *Childhood Executive Functioning Inventory (CHEXI)* *Subscale Regulation, Results of the Longitudinal Analyses of Covariance at the Second and Third Measurement Point* | | | | | | |
| --- | --- | --- | --- | --- | --- | --- |
|  | **T2** | | | **T3** | | |
| Variables | *B (SE)* | 95% CI | *p* | *B (SE)* | 95% CI | *p* |
| (Constant) | -0.87 (0.74) | -2.33 – 0.59 | 0.243 | -0.33 (0.87) | -2.05 – 1.38 | 0.701 |
| Baseline | 0.87 (0.04) | 0.79 – 0.95 | **<0.001** | 0.79 (0.05) | 0.70 – 0.89 | **<0.001** |
| Study Group | -0.18 (0.08) | -0.33 – -0.02 | **0.030** | -0.08 (0.09) | -0.27 – 0.10 | 0.386 |
| Gender | 0.01 (0.08) | -0.16 – 0.17 | 0.951 | 0.07 (0.10) | -0.12 – 0.26 | 0.478 |
| Age | 0.17 (0.10) | -0.04 – 0.37 | 0.110 | 0.08 (0.12) | -0.16 – 0.32 | 0.516 |
| *N* | 205 | | | 200 | | |
| *R*^2^ / *R*^2^ adjusted | 0.709 / 0.703 | | | 0.587 / 0.578 | | |
| *d* [95% CI] | -0.306 [-0.584 – -0.0283] | | | -0.123 [-0.403 – 0.157] | | |
| *Note.* CI = Confidence Interval, Coding Study Group: 1 = Intervention, 0 = Waiting Control Group (Reference), Coding Gender: 1 = Female, 2 = Male. | | | | | | |

| **Table 14**  *Childhood Executive Functioning Inventory (CHEXI)* *Subscale Inhibition, Results of the Longitudinal Analyses of Covariance at the Second and Third Measurement Point* | | | | | | |
| --- | --- | --- | --- | --- | --- | --- |
|  | **T2** | | | **T3** | | |
| Variables | *B (SE)* | 95% CI | *p* | *B (SE)* | 95% CI | *p* |
| (Constant) | -0.31 (0.58) | -1.45 – 0.82 | 0.587 | -0.04 (0.64) | -1.30 – 1.21 | 0.944 |
| Baseline | 0.92 (0.04) | 0.84 – 0.99 | **<0.001** | 0.89 (0.04) | 0.81 – 0.98 | **<0.001** |
| Study Group | -0.16 (0.06) | -0.28 – -0.03 | **0.014** | -0.11 (0.07) | -0.25 – 0.02 | 0.097 |
| Gender | 0.07 (0.07) | -0.06 – 0.20 | 0.311 | -0.00 (0.07) | -0.14 – 0.14 | 0.986 |
| Age | 0.06 (0.08) | -0.10 – 0.22 | 0.443 | 0.03 (0.09) | -0.15 – 0.20 | 0.772 |
| *N* | 205 | | | 200 | | |
| *R*^2^ / *R*^2^ adjusted | 0.763 / 0.759 | | | 0.709 / 0.703 | | |
| *d* [95% CI] | -0.348 [-0.626 – -0.0702] | | | -0.236 [-0.517 – 0.044] | | |
| *Note.* CI = Confidence Interval, Coding Study Group: 1 = Intervention, 0 = Waiting Control Group (Reference), Coding Gender: 1 = Female, 2 = Male. | | | | | | |

| **Table 15**  *Student-Teacher-Relationship Scale (STRS)* *Subscale Closeness, Results of the Longitudinal Analyses of Covariance at the Second and Third Measurement Point* | | | | | | |
| --- | --- | --- | --- | --- | --- | --- |
|  | **T2** | | | **T3** | | |
| Variables | *B (SE)* | 95% CI | *p* | *B (SE)* | 95% CI | *p* |
| (Constant) | 0.94 (0.53) | -0.10 – 1.98 | 0.075 | 1.21 (0.59) | 0.05 – 2.37 | **0.041** |
| Baseline | 0.76 (0.04) | 0.68 – 0.85 | **<0.001** | 0.75 (0.05) | 0.66 – 0.85 | **<0.001** |
| Study Group | 0.14 (0.05) | 0.04 – 0.25 | **0.007** | 0.15 (0.06) | 0.03 – 0.27 | **0.013** |
| Gender | -0.08 (0.06) | -0.19 – 0.03 | 0.139 | 0.00 (0.06) | -0.12 – 0.13 | 0.948 |
| Age | 0.03 (0.07) | -0.10 – 0.17 | 0.634 | -0.02 (0.08) | -0.17 – 0.13 | 0.772 |
| *N* | 205 | | | 200 | | |
| *R*^2^ / *R*^2^ adjusted | 0.646 / 0.639 | | | 0.582 / 0.573 | | |
| *d* [95% CI] | 0.381 [0.102 – 0.660] | | | 0.357 [0.074 – 0.639] | | |
| *Note.* CI = Confidence Interval, Coding Study Group: 1 = Intervention, 0 = Waiting Control Group (Reference), Coding Gender: 1 = Female, 2 = Male. | | | | | | |

| **Table 16**  *Student-Teacher-Relationship Scale (STRS)*  *Subscale Conflict, Results of the Longitudinal Analyses of Covariance at the Second and Third Measurement Point* | | | | | | |
| --- | --- | --- | --- | --- | --- | --- |
|  | **T2** | | | **T3** | | |
| Variables | *B (SE)* | 95% CI | *p* | *B (SE)* | 95% CI | *p* |
| (Constant) | 0.80 (0.47) | -0.12 – 1.73 | 0.088 | 0.08 (0.62) | -1.14 – 1.30 | 0.893 |
| Baseline | 0.74 (0.04) | 0.66 – 0.83 | **<0.001** | 0.80 (0.06) | 0.69 – 0.92 | **<0.001** |
| Study Group | -0.01 (0.05) | -0.11 – 0.09 | 0.829 | -0.02 (0.07) | -0.15 – 0.11 | 0.789 |
| Gender | 0.06 (0.05) | -0.04 – 0.17 | 0.235 | 0.01 (0.07) | -0.13 – 0.15 | 0.903 |
| Age | -0.07 (0.07) | -0.20 – 0.06 | 0.264 | 0.03 (0.09) | -0.14 – 0.20 | 0.752 |
| *N* | 205 | | | 200 | | |
| *R*^2^ / *R*^2^ adjusted | 0.627 / 0.619 | | | 0.516 / 0.507 | | |
| *d* [95% CI] | -0.030 [-0.306 – 0.246] | | | -0.038 [-0.318 – 0.242] | | |
| *Note.* CI = Confidence Interval, Coding Study Group: 1 = Intervention, 0 = Waiting Control Group (Reference), Coding Gender: 1 = Female, 2 = Male. | | | | | | |
